# Multimodality detection of early relapse in radically treated high-risk HPV-negative head and neck squamous cell carcinoma: study protocol for a prospective multicentre UK cohort study (HERD)

**DOI:** 10.1136/bmjopen-2026-121924

**Published:** 2026-09-24

**Authors:** Sanjena Mithra, Ahmed Abdullah Ahmed, Amit Roshan, Paul R Barber, Camila de Carvalho Gomes, Ton Coolen, Elinor Sawyer, Nitzan Rosenfeld, Ralph Sinkus, Miguel Reis-Ferreira, Steve Connor, Karin Shmueli, Simon Morley, Pablo Nenclares, Caroline Apsey, Allan Hackshaw, Asit Arora, Teresita Beeston, Juel Tuazon, Michelle Berin, Antonia Yeung, Chris Curtis, Kenrick Ng, Rami Mustapha, Cheryl Gillett, Balqiisa Issa, Chris Brew-Graves, Maria Lioumi, Tony Ng, Martin Forster

**Affiliations:** 1 University College London, London, UK; 2 Department of Biomedical Engineering and Imaging Science, King’s College London, London, UK; 3 Comprehensive Cancer Centre, King’s College London, London, UK; 4 Barts Cancer Institute Centre for Cancer Cell and Molecular Biology, London, UK; 5 Cancer Research UK Cambridge Research Institute, Cambridge, UK; 6 Department of Plastic & Reconstructive Surgery, Cambridge University Hospitals NHS Foundation Trust, Cambridge, UK; 7 King’s College London, London, UK; 8 Saddle Point Science, York, UK; 9 School of Cancer and Pharmaceutical Sciences, King’s College London Faculty of Life Sciences and Medicine, London, UK; 10 Guy's and St Thomas’ Hospitals NHS Trust, London, UK; 11 Neuroradiology, Guy's and St Thomas’ Hospitals NHS Trust, London, UK; 12 Department of Medical Physics and Biomedical Engineering, University College London, London, UK; 13 University College London Hospitals NHS Foundation Trust, London, UK; 14 Barts and The London NHS Trust, London, UK; 15 CR UK & UCL Cancer Trials Centre, London, UK; 16 The Swallows Head and Neck Cancer Charity, Blackpool, UK; 17 Department of Medical Oncology, St Bartholomew’s Hospital, London, UK; 18 King’s Health Partners Cancer Biobank, King’s College London, London, UK; 19 UCL, London, UK; 20 Division of Surgery and Interventional Science, University College London, London, UK; 21 UCL Cancer Institute, London, UK; 22 Department of Cancer Services, University College London Hospitals NHS Foundation Trust, London, UK

**Keywords:** Head & neck tumours, Head & neck imaging, IMMUNOLOGY, Genomic Medicine, Early Detection of Cancer, Machine Learning

## Abstract

**Introduction:**

Head and neck squamous cell carcinoma (HNSCC) is the seventh most common cancer globally, with over 12 000 new cases annually in the UK. Despite aggressive radical treatment, approximately 50% of patients with locally advanced (stage III/IV), human papilloma virus (HPV)-negative disease relapse within 2 years, often in the first year. Although current post-treatment follow-up schedules are recommended by clinical guidelines, imaging surveillance is led by symptoms after the initial 3 months and reliable biomarkers for early relapse are lacking. Consequently, recurrences are frequently detected too late for surgical salvage, though timely surgery can improve survival by up to 73% in selected cases. Head and Neck Early Relapse Detection Study represents a large-scale, prospective effort to integrate imaging, genomic, immunologic and microbiome datasets for relapse risk stratification and post-treatment surveillance in this high-risk patient group.

**Methods and analysis:**

Design: Prospective, observational cohort study.

Setting: Secondary and tertiary NHS hospitals in the UK.

Participants: Recruiting up to 200 patients with stage III/IV (high-risk) HPV-negative HNSCC. Participants are enrolled into discovery (n=100) and validation (n=100) cohorts. Patients with HPV-positive tumours, metastatic disease or other contraindications defined in the protocol will be excluded.

Interventions: Observational study with no experimental intervention. Participants are followed for 2 years with longitudinal collection of blood, saliva, urine, stool, fresh and archival tumour tissue and advanced MRI where possible in addition to their standard of clinical care.

Primary outcomes: Development and independent validation of a multimodal recurrence risk prediction model.

Secondary outcomes: Identifying patient populations for primary treatment stratification, establishment of a longitudinal biorepository and identifying biological mechanisms of recurrence. Multimodal data will be integrated using machine learning and Bayesian modelling.

**Ethics and dissemination:**

Ethical approval was granted by the Bromley Research Ethics Committee (21/PR/1581). Findings will be disseminated through peer-reviewed publications, conference presentations and scientific meetings.

**Trial registration number:**

NCT05097625.

**Strengths and limitations of this study**

- Multimodal integration of imaging, genomic, immunological and microbiome data aims to enable comprehensive assessment of recurrence risk and underlying biology in high-risk human papilloma virus-negative head and neck squamous cell carcinoma.
- A prospective, multicentre design with independent validation supports generalisability and reduction of overfitting.
- Longitudinal sampling may lead to missing data and variable follow-up completeness.
- Resource-intensive imaging and multi-omics approaches may limit scalability in routine clinical practice.

## Introduction

 Head and neck squamous cell carcinoma (HNSCC) is a highly aggressive malignancy associated with substantial morbidity and mortality. Patients with human papilloma virus (HPV)-negative HNSCC represent 60%–75% of the HNSCC population^1^ and experience substantially poorer outcomes and higher rates of recurrence (ranging from 28% to 67%^2 3^) than those with HPV-associated disease and therefore represent a population most likely to benefit from improved risk-stratified surveillance. Approximately 30% of patients experience recurrence within the first year after radical treatment. Recurrence primarily manifests at the local site or draining lymph nodes in approximately 40% of patients,^3^ with the majority occurring within the first 2 years (~60% within the first year and ~80% within 2 years).^4^ Distant metastasis occurs in up to 20% of patients, and real-world data indicate that up to 90% of patients with metastatic disease succumb within 12 months.^5 6^

Current international guidelines recommend structured post-treatment surveillance following curative treatment for HNSCC, with regular clinical review and flexible endoscopic examination every 2–3 months during the first 2 years, when recurrence risk is greatest, followed by less frequent review thereafter.^7 8^ Baseline cross-sectional imaging is recommended approximately 3 months after completion of treatment to assess treatment response, with fluorodeoxyglucose-positron emission tomography (FDG-PET)/CT specifically recommended following chemoradiotherapy for selected patients with node-positive disease. Beyond this initial assessment, however, surveillance imaging is generally reserved for patients with suspicious symptoms or abnormal clinical findings. Although CT, MRI and FDG-PET/CT are all used in surveillance, there remains no clear consensus regarding the optimal imaging modality, frequency or duration of routine imaging in asymptomatic patients, and practice varies between institutions.^7^ Consequently, recurrent disease may only become clinically apparent once symptoms develop, reducing opportunities for potentially curative salvage treatment. These limitations highlight the need for validated biomarkers to support personalised, risk-stratified surveillance and earlier detection of relapse.

The Head and Neck Early Relapse Detection (HERD) Study aims to address this through a prospective, multimodal approach to recurrence prediction in high-risk (stage III/IV) HPV-negative HNSCC. It integrates advanced multiparametric MRI, immune profiling, tumour-informed circulating tumour DNA (ctDNA), genomic analysis, exosomal biomarkers, patient-derived organoids (PDOs) and microbiome profiling to characterise complementary aspects of tumour biology. These multimodal datasets are integrated using machine learning and Bayesian modelling to develop a predictive recurrence risk signature (figure 1). Here, we describe the protocol for the ongoing HERD study (Protocol V.5.0, 25 July 2025).

**Figure 1:**
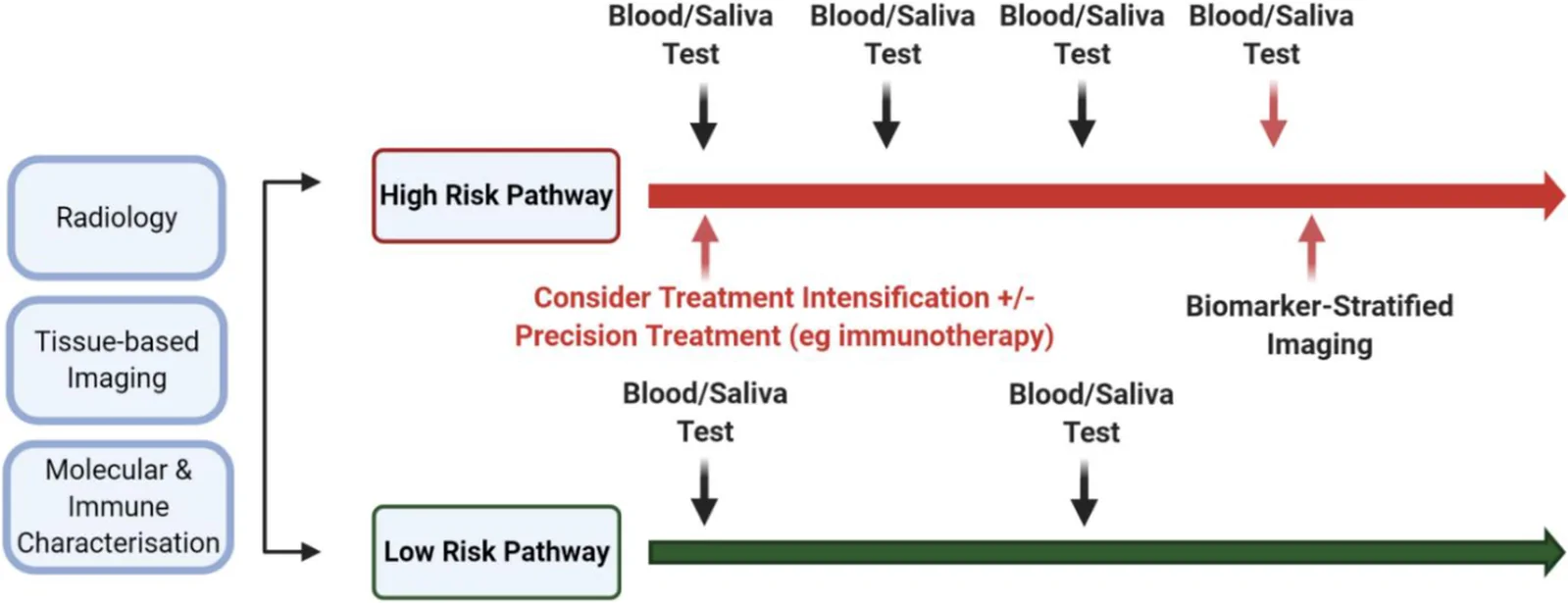
Risk-stratified surveillance pathways and non-invasive surveillance strategy. There is an unmet clinical need to better risk-stratify patients with HNSCC undergoing primary radical treatment. This stratification would benefit high-risk patients by facilitating treatment intensification and utilisation of an integrated, multidimensional surveillance programme capable of detecting dynamic biomarkers predictive of recurrence. Conversely, lower-risk patients may be suited for less intensive monitoring.

### Background and hypotheses

Despite advances in surgery, radiotherapy and systemic therapy, recurrence remains a major cause of mortality in HNSCC. No single biomarker adequately captures the biological complexity of recurrence, which is driven by multiple interconnected processes including tumour hypoxia,^9^ stromal remodelling,^10^ residual disease,^11^ immune evasion and tumour-host interactions.^12^ Current risk stratification relies largely on anatomical and histopathological features and has limited ability to predict relapse. Although p16 immunohistochemistry is a validated surrogate prognostic marker for HPV-driven oropharyngeal squamous cell carcinoma, it does not reliably reflect oncogenic drivers or recurrence risk in other HNSCC subsites.^13^ There is therefore a need for complementary biomarkers that reflect these distinct biological mechanisms and can be integrated into a multimodal prediction framework.

Local recurrence is driven by complex and reciprocal interactions between tumour cells and the tumour microenvironment (TME) that enable immune evasion, persistence of residual disease and expansion of invasive clones despite aggressive and seemingly successful initial therapy.^11^ Lymphovascular invasion (LVI) is a key contributor and is shaped by immune and stromal components of the TME. Pro-angiogenic Tie-2-expressing macrophages facilitate tumour cell intravasation and vascular remodelling, while RORγt^+^ type 3 innate lymphoid cells modulate stromal organisation and T-cell responses, together creating a permissive niche for tumours to thrive.^14–16^

Studies, including a Phase 2 trial by Ng *et al*,^17^ suggest that analysing exosomal and immune parameters in peripheral blood can help monitor treatment responses and predict recurrence risk, thereby aiding personalised surveillance strategies. Stromal remodelling and extracellular matrix deposition are increasingly recognised as regulators of tumour invasion and therapeutic resistance.^18^ Collagen synthesis in hypoxic tumours is driven in part by the hypoxia-inducible enzyme prolyl-4-hydroxylase alpha-1 (P4HA1), and detection of circulating exosomal P4HA1 in patients with HNSCC suggests its potential as a minimally invasive surrogate of tumour hypoxia and stromal activity.^9 19^

Tumour hypoxia is a major determinant of radioresistance and recurrence in HNSCC, irrespective of treatment modality.^20 21^ Complementary imaging techniques, including dynamic contrast-enhanced MRI (DCE-MRI), which evaluates tumour perfusion,^21^ and quantitative susceptibility mapping (QSM),^22^ which provides an indirect measure of tissue oxygenation, offer non-invasive assessment of the hypoxic TME.^20 23–25^ In parallel, MR elastography (MRE) quantifies tissue shear stiffness and viscosity, providing imaging biomarkers of stromal remodelling, extracellular matrix organisation and intratumoural pressure.^26 27^ These biomechanical parameters serve as surrogate markers of cancer aggressiveness and LVI, providing a biological rationale for their inclusion in recurrence prediction models.^28^

Liquid biopsy approaches in HNSCC using serial plasma or saliva sampling have demonstrated good correlation with tumour burden and disease outcome but often need tumour-informed approaches to reach the parts-per-million sensitivity needed for accurate prediction.^29^ TP53 mutations are highly prevalent in HNSCC, and p53-mutated tumour cells release exosomes containing specific microRNAs that reprogramme tumour-associated macrophages towards immunosuppressive phenotypes, illustrating tumour-immune crosstalk.^30–32^

Tumour-derived genetic alterations detected in circulating cell-free DNA (cfDNA) and exosomes provide minimally invasive markers of residual disease and have shown promise for identifying molecular recurrence before clinical relapse. In parallel, immune dysregulation, including expansion of immunosuppressive cell populations such as Interleukin (IL)-10-producing B and T lymphocytes, promotes immune evasion and suppression of anti-tumour immune responses, making these populations attractive candidate biomarkers of recurrence.^33^

Although surgery, radiotherapy and systemic therapy can achieve locoregional tumour control, they may also remodel stromal and immune compartments in ways that are incompletely understood. In preclinical models, certain cytotoxic agents remodel the TME by mobilising bone marrow-derived immune cells, including myeloid-derived suppressor cells and proangiogenic macrophages, contributing to an immunosuppressive landscape.^33 34^ The relevance in HNSCC remains uncertain, underscoring the need to characterise longitudinal treatment-induced microenvironmental changes.

PDOs provide an ex vivo model that preserves many of the biological characteristics of the original tumour, enabling functional investigation of tumour biology, therapeutic response and TME interactions. Within HERD, PDOs will provide a complementary translational platform to validate imaging and molecular biomarkers and support mechanistic studies.

Finally, the oral microbiome represents an additional modulator of tumour immunity.^35^ Fusobacterium nucleatum (which is linked to colorectal cancer) and other oral bacteria can bind tumour-associated glycans and immune checkpoint receptors,^36^ influencing immune activation and chemotherapy responses,^37^ providing rationale for incorporating saliva, stool and urine microbiome profiling into recurrence risk modelling.

Together, these biological mechanisms provide the rationale for a multimodal approach to recurrence prediction. HERD therefore evaluates biomarkers that capture each of these biological domains using complementary imaging, molecular and functional platforms, to evaluate their combined ability to identify patients at increased risk of relapse. The study hypotheses are outlined below.

#### Hypothesis 1: integrated imaging and exosome profiling of tumour hypoxia and stromal remodelling identifies patients at increased risk of recurrence

We will evaluate:

1. Quantitative DCE-MRI perfusion parameters and QSM-derived susceptibility measurements as complementary imaging biomarkers of tumour hypoxia.
2. Exosomal P4HA1 as a circulating marker of hypoxia-associated extracellular matrix remodelling.^38^
3. MRE derived shear stiffness and viscosity as imaging biomarkers of stromal remodelling and LVI.

#### Hypothesis 2: tumour genetic alterations and systemic immune profiles together capture TME interactions that drive HNSCC recurrence

We will:

1. Characterise genomic alterations, including TP53 mutations, using whole genome sequencing (WGS) of tumour tissue together with genomic analysis of paired plasma and saliva cfDNA and exosomes.
2. Characterise systemic and tumour immune phenotypes using flow cytometry, CyTOF and high-plex immunofluorescence, with particular focus on IL-10-producing B-cell and T-cell populations.
3. Use PDOs, where available, to investigate how genomic and immune characteristics relate to tumour biology and treatment response.

#### Hypothesis 3: tumour-associated microbiota modulate immune cell infiltration and treatment response, thereby influencing recurrence risk in HNSCC

We will:

1. Use 16S rRNA sequencing to evaluate their association with recurrence.
2. Investigate microbiome/tumour interactions using tumour-immune co-culture systems and complementary PDO models, alongside three-dimensional imaging approaches, to explore mechanisms underlying immune modulation and treatment response.

### Objectives

The purpose of this study is to:

- Develop and validate a minimally invasive multi-analyte based risk signature to enhance detection of early relapse of radically treated HPV-negative HNSCC.
- Stratify recurrence risk within this high-risk cohort using longitudinal assessment of minimally invasive biomarkers.

Key secondary objectives include:

- Characterising imaging, molecular, immune and microbiome biomarkers associated with recurrence in HPV-negative HNSCC.
- Identifying patients who would benefit from individualised discussions regarding treatment options, including the potential for treatment intensification, biomarker-driven precision medicine and enrolment in clinical trials.
- Developing a comprehensive head and neck cancer biorepository, integrating radiological, tissue-based imaging, molecular and immune profiles, to support future translational research.
- Identifying novel biomarkers, particularly within the high-risk group, to uncover new therapeutic targets and inform future treatment strategies.

### Study status and regulatory milestones

Funding was achieved through a grant awarded by Cancer Research UK (CRUK) Early Detection and Diagnosis Committee (EDDCPGM\100001) on 14 July 2021. The HERD clinical study was registered on Clinical Trials (NCT05097625) on 28 October 2021 and ethical approval was granted by the Bromley Research Ethics Committee (21/PR/1581) on 8 December 2021. Recruitment opened in May 2023 following delays due to contracting, imaging implementation and legacy impacts of the COVID-19 pandemic. These delays prompted four substantial amendments (SA01—January 2023, SA02 and SA03—June 2024, SA04—March 2025) to broaden eligibility criteria while maintaining the scientific objectives of the study and open additional recruiting centres (shown in table 1). In addition, CRUK approved a no-cost extension until February 2028 to facilitate completion of recruitment. Recruitment is ongoing, with additional NHS sites able to be activated under the same approved protocol, harmonised imaging procedures, laboratory standard operating procedures (SOPs) and centralised data management. At the time of writing, 54 participants have been enrolled.

**Table 1:** Key eligibility criteria*

Inclusion criteria	Exclusion criteria
Histologically confirmed, stage III/IV HNSCC, HPV negative	HPV positive disease
Offered radical treatment (This includes primary surgery and any postoperative adjuvant/consolidation treatment or primary radical (±chemo) radiotherapy)	Participants with concurrent malignancy
Age ≥18 years	Confirmed distant metastatic disease
Adequate bone marrow function (neutrophils, platelets and haemoglobin grade 0 or 1 according to CTCAE)	Known medical history of hepatitis B, C or HIV infection
Able to provide informed consent	Pregnant or lactating patients

Contraindications to MRI (including but not limited to cardiac pacemaker, metallic implants, major claustrophobia) will be taken into account but do not preclude enrolment into the study. Specialised MRE is delivered at Guy’s and UCLH sites only.

* Imaging considerations in determining eligibility.

CTCAE, Common Terminology Criteria for Adverse Events; HNSCC, head and neck squamous cell carcinoma; HPV, human papilloma virus; MRE, magnetic resonance elastography; UCLH, University College Hospitals NHS Trust.

## Methods

### Study design

This is a prospective observational cohort study aiming to recruit 200 participants with newly diagnosed, locally advanced (stage III/IV), HPV-negative HNSCC of the oral cavity, oropharynx, hypopharynx or larynx. Participants will be identified through head and neck multidisciplinary team (MDT) meetings and enrolled as inpatients or outpatients. Participants will undergo one of two standard-of-care treatment pathways (figure 2):

1. Surgical cohort: surgery with radical intent (with or without reconstruction), followed by postoperative (chemo)radiotherapy where indicated.
2. Non-surgical cohort: definitive radical (chemo)radiotherapy.

**Figure 2:**
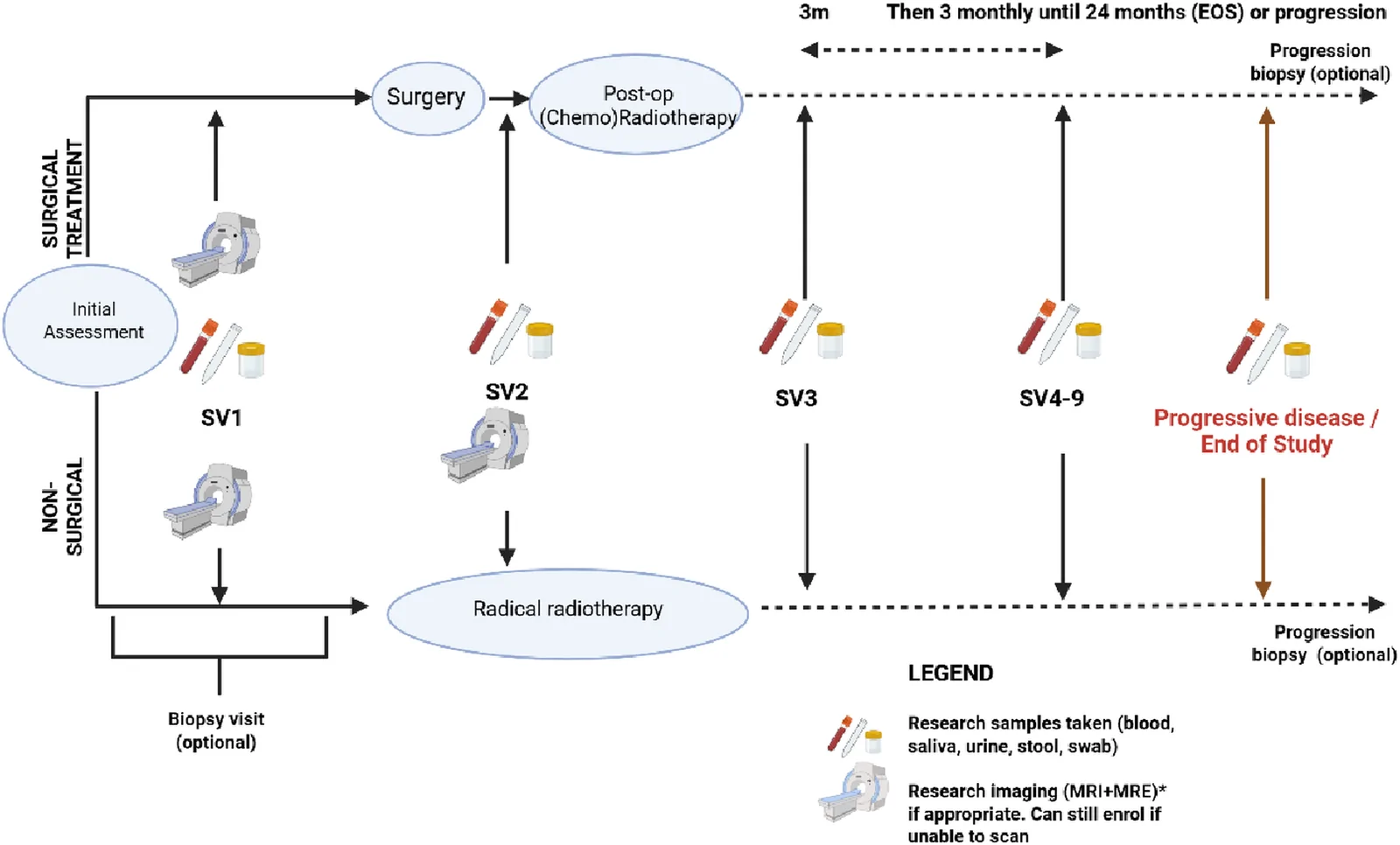
Schematic diagram of the HERD study protocol. EOS, end of study; HERD, Head and Neck Early Relapse Detection; MRE, magnetic resonance elastography; SV, study visit.

Treatment allocation will not be randomised, as this is an observational study, and treatment decisions will be determined by the MDT in accordance with clinical guidelines, reflecting real-world practice. All study-related procedures will be conducted alongside standard clinical care and will not alter treatment delivery.

Blinding is not feasible due to the nature of the interventions and the integration of clinical, imaging and biological data; however, outcome modelling will be conducted using predefined analytical pipelines to minimise bias.

Analyses will be conducted in two stages: a discovery cohort (n=100) for predictive model development and an independent validation cohort (n=100) to assess model performance. This split-sample design will provide an unbiased estimate of predictive accuracy with associated CIs.

All recruited participants will undergo standard-of-care diagnostic tests such as imaging and pre-treatment blood tests. Following consent, additional blood samples will be taken for immune landscape analysis, exosomal protein/microRNA and genetic profiling. Saliva samples and tumour samples from initial diagnostic procedures will be analysed for genetic alterations such as somatic mutations, immune profile and microbiome. Research imaging will also be performed where appropriate (see online supplemental table 2 in Supplementary Material for full study schedule).

### Study setting

Participants will be identified by clinical teams and recruited into this study by the research team in their respective hospitals. They will not be recruited by publicity. HERD is currently open at three London hospitals: Guys & St Thomas’ NHS Trust, University College London (UCL) NHS Trust and Barts & The London NHS Trust. Any additional recruiting sites will be activated by using the same SOPs to ensure consistency across sites.

### Study considerations

#### Variables

Key variables within the HERD study include biological exposures (MRI-derived biomarkers, tumour mutations, immune profiles and microbiome features) that inform recurrence prediction. Clinical predictors such as age, tumour site and treatment modality will be incorporated into risk modelling. Potential confounders, including smoking status, alcohol use, comorbidities and treatment factors, will be adjusted for to minimise bias. Effect modification (eg, by immune phenotype or p53 status) will be explored to ensure model applicability across patient subgroups. The statistical modelling framework, incorporating machine learning and Bayesian approaches, will implement structured feature selection, adjustment and interaction modelling to enhance robustness and generalisability.

#### Bias

Potential sources of bias include measurement variability in imaging and biomarker assays, confounding by clinical characteristics and observer-dependent variability in imaging or immune profiling and variability between recruiting centres. These risks will be mitigated through standardised protocols, predefined operating procedures across sites and appropriate statistical adjustment within the modelling framework. Where appropriate, recruiting centre will be explored in sensitivity analyses.

### Laboratory analyses

Genomic profiling strategies will include WGS on tumour tissue alongside genomic analysis of paired plasma and saliva cfDNA, while immune profiling will incorporate flow cytometry, CyTOF and high-plex immunofluorescence. Microbiome profiling will be performed using 16s RNA sequencing of saliva, stool and urine samples. PDOs will be established from fresh surgical tumour tissue where available and undergo histopathological, molecular and functional characterisation. Where appropriate, tumour-immune co-culture assays will be performed to investigate TME interactions. These procedures have been standardised across participating sites by the relevant work package leads to ensure consistency in sample processing, quality assurance and data generation. As this manuscript describes the clinical study design, detailed laboratory methodologies are not presented here but will be reported in full alongside the corresponding translational analyses.

### Statistical methods

No formal a priori power calculation was performed because HERD is a multimodal prediction model development study rather than a conventional hypothesis-testing study. For this type of high-dimensional multivariate analysis, there is no straightforward method for calculating statistical power based on the anticipated numbers of covariates, participants, censoring and missing data. Instead, the target sample size of 200 participants (100 discovery, 100 validation) was selected a priori based on the planned analytical framework. Prior simulation studies of the Bayesian modelling pipeline demonstrated robust predictive performance using independent training and validation datasets of this size, supporting the proposed discovery/validation design.^39 40^ Participants will be assigned to the discovery or validation cohort according to recruitment sequence, with recruitment continuing across all participating sites throughout both phases.

The number of covariates across imaging, molecular, immune and microbiome platforms is expected to exceed the number of participants in the discovery cohort (n=100), but our analytical strategy is designed to address this dimensionality challenge. Rather than applying standard regression approaches directly to all covariates, we will implement a two-stage inference framework (figure 3). In the first stage, biologically related variables are integrated into a small number of mechanistic and platform-level meta-covariates. By reducing dimensionality at each modelling stage, the number of effective predictors remains substantially below the number of training samples (anticipated ≤80), reducing the risk of overfitting. The resulting representation is used to generate an overall recurrence risk predictor, allowing us to probe synergistic interactions between mechanistic processes or modalities. This approach is consistent with reported multimodal data-fusion frameworks that integrate heterogeneous biological measurements into joint predictive models.^41^ The analysis platform, developed in collaboration with Saddle Point Science Ltd (https://www.saddlepointscience.co.uk/), builds in safeguards against overfitting that are particularly important for high-dimensional data with small-sample sizes. These include regularisation methods, structured preprocessing workflows and diagnostic checks to detect unstable models.^42^ The independent validation cohort (n=100) provides an unbiased estimate of predictive performance with associated CIs, reducing optimism bias in model development.

**Figure 3:**
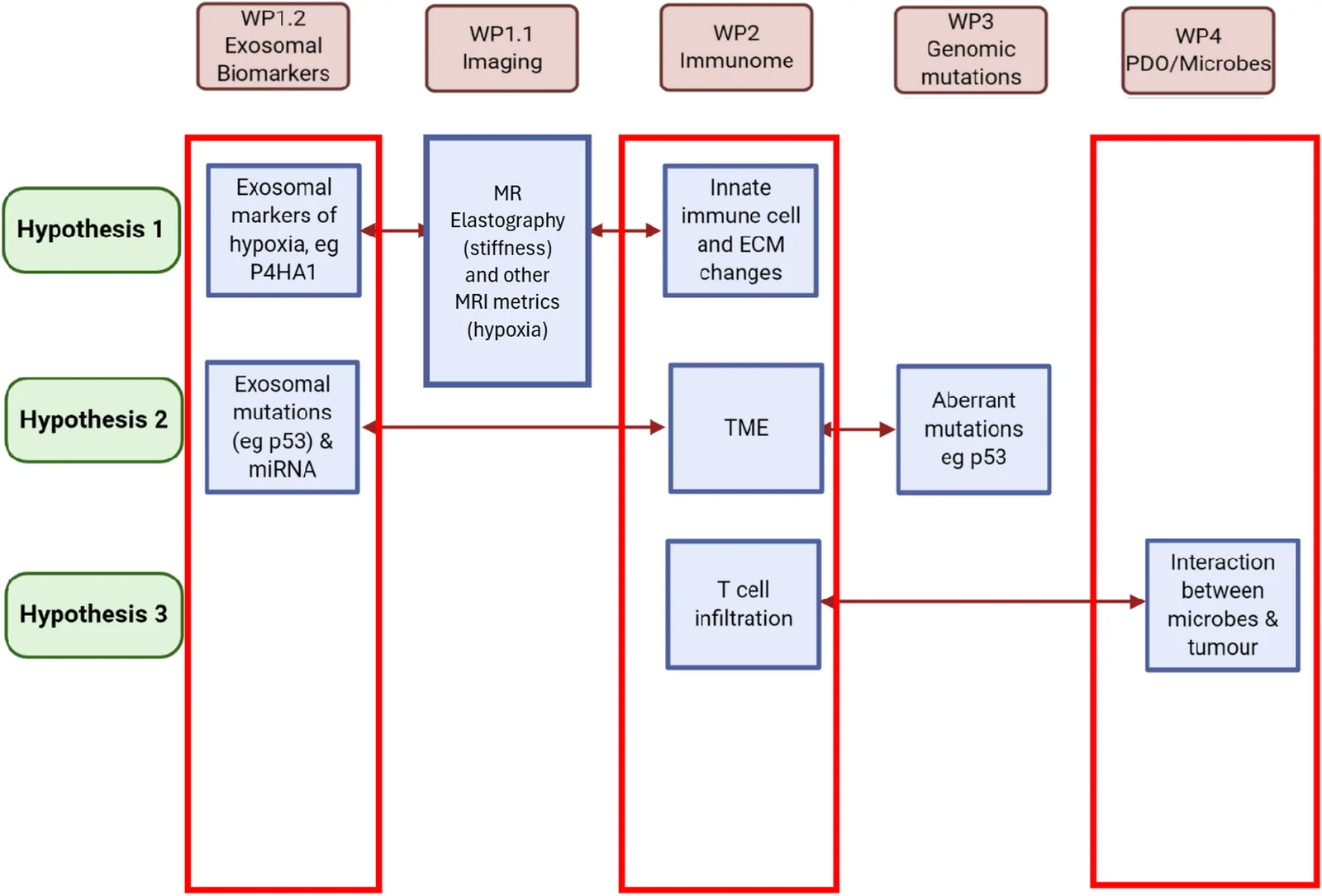
Integrated risk model. This figure demonstrates the interconnectivity between multiple detection tools/modalities for construction of an integrated risk diagnostic/model. Three main biological hypotheses (H1 - H3, described above) will be addressed through a number of inter-connected technology work package platforms (WP1 - WP5). Each of the linkages (horizontal bidirectional arrows in the lower panel) is comprised of parameters measured in the different technological modalities - together these linked parameters make up mechanistic meta-covariates (WP5) for our mathematical model analysis. ECM, extracellular matrix; P4HA1, prolyl-4-hydroxylase alpha-1; PDO, patient-derived organoid; TME, tumour microenvironment.

The analytical framework has been benchmarked using realistic synthetic datasets that reproduce common challenges in clinical data, including missing values, patient heterogeneity and competing risks.^39^ These simulations enable systematic comparison of regression algorithms and estimation of expected error rates, providing a framework to assess model robustness and interpretability. Missing data will be assessed for mechanism and handled using appropriate methods (eg, multiple imputation), with sensitivity analyses performed to assess impact on model performance.

### Summary

HERD is a prospective multimodal translational study investigating biological mechanisms and biomarkers associated with recurrence in high-risk HPV-negative HNSCC.

It integrates imaging, liquid biopsy, immune profiling and microbiome analysis within a unified computational framework to better characterise TME interactions that underlie relapse. Recruitment to complex multimodal translational studies is inherently challenging and expansion to additional recruiting centres may introduce variability; however, harmonised protocols, SOPs and centralised quality assurance have been implemented to minimise centre-related heterogeneity.

HERD’s multicentre design increases the diversity of the study population and supports evaluation of proposed biomarkers across multiple NHS settings. This is so findings are not limited to a specific institution and more likely to be applicable to a broader population. Integration of exosomal biomarkers, multiparametric MRI, immune phenotyping and microbial profiling enables interrogation of complementary biological processes, including hypoxia, stromal remodelling, tumour genomics and immune modulation. This systems-level approach is intended to capture the multifactorial drivers of recurrence more effectively than single-modality biomarkers.

A key methodological strength is the structured analytical framework designed to address high-dimensional data relative to sample size. This pipeline is designed to reduce overfitting effects and improve model robustness in high-dimensional predictive modelling. This is particularly important when dealing with large datasets with multiple variables that could otherwise lead to skewed or non-generalisable results.

The study focuses on a clinically high-risk population with limited validated relapse biomarkers, representing an important unmet clinical need. However, several limitations warrant consideration. As an observational study, causal inference will be limited and the resource-intensive nature of advanced imaging, multi-omics profiling and PDO generation may restrict scalability in lower-resource settings. In addition, longitudinal sampling and imaging requirements introduce logistical complexity and may affect adherence and data completeness. Despite these challenges, HERD provides a prospective framework for integrating imaging, molecular and functional biomarkers to investigate recurrence in high-risk HPV-negative HNSCC.

### Study monitoring

HERD is conducted in compliance with Medicines and Healthcare products Regulatory Agency regulatory requirements, Research Ethics Committee (REC) approval and the Sponsor’s (UCL) approval; represented by National Cancer Imaging Translational Accelerator (NCITA) SOPs. A tailored database system is used for the purposes of the study and securely stored in the NCITA UCL online repository. The Trial Management Group and Trial Steering Committee represent a variety of disciplines who oversee data monitoring and outcome evaluation.

### Patient and public involvement

Patient-facing materials, including the Patient Information Sheet and Informed Consent Form, were developed with input from the founder of The Swallows Head & Neck Cancer Charity (https://theswallows.org.uk/). Written informed consent will be obtained from all participants prior to enrolment. Study findings will be shared with participants through The Swallows Head & Neck Cancer Charity and appropriate patient-facing summaries following publication.

### Sample handling

Tissue and all other samples will be collected from participants and stored (after analysis in HERD) for future translational work via the Kings Health Partners Biobank—REC 23/EE/0005, Health Research Authority (HRA) storage license: 12121 and the UCL Cancer Institute Biobank—REC 20/YH/0088, HRA storage license: 12055. Participants will be consented for both HERD and the respective biobank.

### Safety reporting

As this is an observational study with no experimental treatments or interventions, no significant study-related adverse events (AEs) are anticipated. Any AE arising from routine research procedures (eg, blood sampling, contrast administration) will be recorded and reported in accordance with institutional policies and sponsor requirements within 5 days. Incidental findings in this study are an unlikely event, as the scope of imaging is limited to a defined anatomical area of interest which would have been routinely scanned, that is, the head and neck region. However, any abnormalities identified on research imaging will be highlighted by the reporting radiologist to the research team who will notify the site principal investigator. They will in turn alert the participant’s clinical team.

## Ethics and dissemination

Ethical approval was obtained from the National Research Ethics Committee (21/PR/1581). Results will be disseminated via peer-reviewed publications.

We used Strengthening the Reporting of Observational Studies in Epidemiology (STROBE) reporting guidelines^43^ to draft this manuscript, and the STROBE reporting checklist^44^ when editing, included in online supplemental information.

### Data statement

No datasets have been analysed at the time of publication of this protocol. Data arising from the study will be stored within a secure research repository and may be made available to qualified researchers on reasonable request, subject to approval by the study investigators, relevant ethics committees and data access governance processes.

## Supplementary material

10.1136/bmjopen-2026-121924 online supplemental table 1

10.1136/bmjopen-2026-121924 online supplemental file 1
