# Supplementary material for: Multimodality detection of early relapse in radically treated high-risk HPV-negative head and neck squamous cell carcinoma: study protocol for a prospective multicentre UK cohort study (HERD)
Source: BMJ Open. 2026 Sep 24;16(9):e121924. doi: 10.1136/bmjopen-2026-121924 (PMC13629944; doi:10.1136/bmjopen-2026-121924)
Supplement: Supplementary file 1 [file bmjopen-2026-121924.reviewer_comments.pdf]

PEER REVIEW HISTORY

BMJ Open publishes all reviews undertaken for accepted manuscripts. Reviewers are asked to complete a checklist review form and are provided with free text boxes to elaborate on their assessment. These free text comments are reproduced below.

ARTICLE DETAILS

Title (Provisional)

STUDY PROTOCOL: Multimodality detection of early relapse in radically treated high-risk HPV-negative head and neck squamous cell carcinoma: protocol for a prospective multicentre UK cohort study (HERD)

Authors

Mithra, Sanjena; Ahmed, Ahmed Abdullah Abdullah; Roshan, Amit; Barber, Paul R; de Carvalho Gomes, Camila; Coolen, Ton; Sawyer, Elinor; Rosenfeld, Nitzan; Sinkus, Ralph; Reis-Ferreira, Miguel; Connor, Steve; Shmueli, Karin; Morley, Simon; Nenclares, Pablo; Apsey, Caroline; Hackshaw, Allan; Arora, Asit; Beeston, Teresita; Tuazon, Juel; Berin, Michelle; Yeung, Antonia; Curtis, Chris; Ng, Kenrick; Mustapha, Rami; Gillett, Cheryl; Issa, Balqiisa; Brew-Graves, Chris; Lioumi, Maria; Ng, Tony; Forster, Martin

---

VERSION 1 - REVIEW

**Reviewer**	**1**
**Name**	**Perréard, Marion**
**Affiliation**	**Department of Otolaryngology-Head and Neck Surgery, CHU de Caen**
**Date**	**25-Jun-2026**
**COI**	**None**

This study protocol describes an ambitious study focused on the detection of relapse in high risk HNSCC using multiple modalities.

A- Major remarks:

- 1) Introduction: The current surveillance protocols recommended by guidelines—though debated—are not described. Key details such as the frequency of clinical examinations and the types of imaging performed are missing, as is an explanation of why these protocols may be insufficient.

The statement in the Abstract that “post-treatment surveillance isn’t standardized” is only partially accurate, as guidelines in many countries provide clear recommendations for surveillance strategies.

- 2) Background and Hypotheses: The study examines numerous variables, resulting in a brief description of each readout's background. I would expect a more focused justification, particularly regarding the follow-up expectations and the potential of these prognostic markers as relapse indicators.

The hypotheses introduce several concepts (e.g., DCE perfusion, QSM, IL-10-secreting cells, PDOs) that are not explained earlier in the text.

- 3) Methods: While the clinical design of the study is well described, the protocol relies on multiple fundamental analyses that are not detailed at all. These include genomic signature analysis, the establishment of patient-derived organoids (PDOs), co-culture management…

- 4) Number of participants: Only 48 patients have been enrolled over three years, with an extension planned until 2028. Recruiting 200 patients by then seems challenging. Additionally, the mention of "further UK sites planned" implies that the study will open in other centers during its course, which could introduce bias even more if patients are divided into two successive cohorts of 100.

B- Minor remarks:

- 1) The rationale for focusing on HPV-negative HNSCC patients is presented at the end of the introduction. For clarity, this justification should be introduced earlier.

- 2) References to "g" and "h" in Table 2 are missing.

This study addresses a critical question in HNSCC follow-up: improving relapse detection. While the use of multiple modalities is ambitious, a more rigorous scientific justification for each modality and a clearer description of the readouts are necessary.

VERSION 1 - AUTHOR RESPONSE

Reviewer: 1 Dr. Marion Perréard, Department of Otolaryngology-Head and Neck Surgery, CHU de Caen

Reviewer comment	Response
A1) This study protocol describes an ambitious study focused on the detection of relapse in high risk HNSCC using multiple modalities. This study addresses a critical question in HNSCC follow-up: improving relapse detection. While the use of multiple modalities is ambitious, a more rigorous scientific justification for each modality and a clearer description of the readouts are necessary. write a paragraph to address point 1 and references	We thank the reviewer for this thoughtful comment. We agree that the scientific rationale for the multimodal approach should be more clearly articulated.<br>We have revised the **Background and Hypotheses** section to provide a clearer biological rationale for the multimodal approach, explaining how each imaging, molecular, immunological and functional modality interrogates underlying biological mechanisms of HNSCC recurrence. We have also expanded the descriptions of the planned readouts to improve clarity while remaining appropriate for a clinical study protocol.

	Page 4
A2) Introduction: The current surveillance protocols recommended by guidelines—though debated—are not described. Key details such as the frequency of clinical examinations and the types of imaging performed are missing, as is an explanation of why these protocols may be insufficient. The statement in the Abstract that "post-treatment surveillance isn't standardized" is only partially accurate, as guidelines in many countries provide clear recommendations for surveillance strategies.	We thank the reviewer for this valuable comment and agree that our original wording did not sufficiently distinguish between established clinical follow-up recommendations and surveillance imaging practices. We have revised the Introduction and Abstract to provide a summary of current international surveillance guidelines, including the recommended frequency of clinical review and post-treatment imaging. We have also clarified that, while structured clinical follow-up is well established, there remains uncertainty regarding the optimal frequency, modality and duration of routine surveillance imaging in asymptomatic patients, with variation between institutions and guidelines. We believe this more accurately reflects current practice and better highlights the clinical need addressed by HERD.<br>Page 3
A3) Background and Hypotheses: The study examines numerous variables, resulting in a brief description of each readout's background. I would expect a more focused justification, particularly regarding the follow-up expectations and the potential of these prognostic markers as relapse indicators. The hypotheses introduce several concepts (e.g., DCE perfusion, QSM, IL-10-secreting cells, PDOs) that are not explained earlier in the text.	We thank the reviewer for this helpful suggestion. We have substantially revised the **Background and Hypotheses** section to provide a clearer biological rationale for each modality and to better link the selected biomarkers to mechanisms underlying recurrence. We have expanded the descriptions of tumour hypoxia, stromal remodelling, immune dysregulation and TME interactions, and introduced concepts such as DCE-MRI, QSM, IL-10-secreting immune cells and patient-derived organoids (PDOs) before they appear within the study hypotheses. We have also refined the hypotheses to emphasise the complementary biological processes being investigated rather than the technologies themselves, improving the overall coherence and rationale of the study design.<br>Page 4-6
A4) Methods: While the clinical design of the study is well described, the protocol relies on multiple fundamental analyses that are not detailed at all. These include genomic signature analysis, the establishment of patient-derived organoids (PDOs), co-culture management…	We thank the reviewer for this important comment. We agree that the manuscript should clarify how these translational analyses will be undertaken and have therefore expanded the descriptions of the planned readouts, including whole genome sequencing, immune phenotyping (flow cytometry, CyTOF and high-plex immunofluorescence), microbiome profiling, exosome characterisation and PDO generation, to provide greater clarity while remaining within the scope of a clinical study

	protocol. Detailed laboratory methodologies will be reported separately alongside the relevant translational analyses.<br>Page 13
A5) Number of participants: Only 48 patients have been enrolled over three years, with an extension planned until 2028. Recruiting 200 patients by then seems challenging. Additionally, the mention of "further UK sites planned" implies that the study will open in other centers during its course, which could introduce bias even more if patients are divided into two successive cohorts of 100.	We thank the reviewer for this important observation. We acknowledge that recruitment to complex prospective translational studies has been slower than originally anticipated, largely due to delays in study activation and implementation of advanced imaging protocols. To address these challenges, the protocol has undergone several amendments to broaden eligibility while maintaining the scientific objectives of the study, additional recruiting sites have been opened, and CRUK has approved a no-cost extension until February 2028. We have revised the manuscript to clarify the recruitment timeline and current study status. We have also clarified that any additional sites will recruit under the same approved protocol, using harmonised eligibility criteria, imaging procedures, laboratory standard operating procedures and centralised data management. Finally, we have added text to explain that discovery and validation cohorts are defined by recruitment sequence rather than recruiting centre, with participants from all sites contributing to both cohorts, thereby minimising the potential for centre-related bias.<br>Page 8, 13, 14
B1) The rationale for focusing on HPV-negative HNSCC patients is presented at the end of the introduction. For clarity, this justification should be introduced earlier.	We thank the reviewer for this suggestion. We have revised the Introduction so that the rationale for focusing on patients with HPV-negative HNSCC is introduced in the opening paragraph, providing earlier clinical justification for the study population.<br>Page 3
B2) References to "g" and "h" in Table 2 are missing.	We thank the reviewer for highlighting this omission. The references to footnotes **g** and **h** have now been added to Table 2.<br>Page 11
