# Supplementary material for: Multimodality detection of early relapse in radically treated high-risk HPV-negative head and neck squamous cell carcinoma: study protocol for a prospective multicentre UK cohort study (HERD)
Source: BMJ Open. 2026 Sep 24;16(9):e121924. doi: 10.1136/bmjopen-2026-121924 (PMC13629944; doi:10.1136/bmjopen-2026-121924)
Supplement: Supplementary file 2 [file bmjopen-16-9-s001.docx]

|  | Screening visit | Sampling visit 1 (SV1) | Treatment visit (0) | Sampling visit 2 (SV2) | 3-month visit (SV3) | 6-month visit (SV4) | 9-month visit (SV5) | 1 year visit (SV6) | 15-month visit (SV7) | 18-month visit (SV8) | 21-month visit (SV9) | 2-year visit  (End of study) / Progression visit (EOS / PV) |
| --- | --- | --- | --- | --- | --- | --- | --- | --- | --- | --- | --- | --- |
|  | Week -2  (+/- 1 week) |  | Baseline Week 0 | Week 2 (+/- 1 week) | Week 12 (+/- 1 week) | 6 months (+/- 4 wks) | 9 months (+/- 4 wks) | 12 months (+/- 4 wks) | 15 months (+/- 4 wks) | 18 months (+/- 4 wks) | 21 months (+/- 4 wks) | 24 months  (+/- 4 wks) |
| Informed Consent | x |  |  |  |  |  |  |  |  |  |  |  |
| ECOG Performance Status | x |  |  | x | x | x | x | x | x | x | x | x |
| Past Medical History | x |  |  |  |  |  |  |  |  |  |  |  |
| Interim Health Assessment^a^ |  |  |  | x | x | x | x | x | x | x | x | x |
| Selected Concomitant Medication^b^ | x |  |  | x | x | x | x | x | x | x | x | x |
| Haematology^c^ | x |  |  | x |  |  |  |  |  |  |  |  |
| Blood Chemistry^c^ | x |  |  | x |  |  |  |  |  |  |  |  |
| Pregnancy Test  (if applicable) | x | |  |  |  |  |  |  |  |  |  |  |
| Tissue Collection (Surgical arm) |  | | X |  |  |  |  |  |  |  |  |  |
| Tissue Biopsy  (Optional/Archival)^d^ | x | |  |  |  |  |  |  |  |  |  | x |
| Tissue Swab^e^ | x | |  |  | x | x |  | x |  |  |  | x |
| Urine Sample | x | |  |  | x | x |  | x |  |  |  | x |
| Stool Sample | x | |  |  | x | x |  | x |  |  |  | x |
| Salivary Tests | x | |  | x | x | x |  | x |  |  |  | x |
| Research bloods^f^ | x | |  | x | x | x | x | x | x | x | x | x |
| Standard Imaging (MRI or CT) | x | |  |  |  |  |  |  |  |  |  |  |
| Research Imaging  (Optional MRI +/- MRE)^g,h,i^ | x | |  | x |  |  |  |  |  |  |  |  |
| Adverse Events | x | |  | x | x | x | x | x | x | x | x | x |

**Table 2. Full Study Schedule**

a. Full systems examination at screening then targeted examination from V2 (vitals not required to be recorded).
b. Selected concomitant medications to be recorded (steroids, antibiotics or analgesia; specifically, opioids) indicating the dose, frequency, duration, and indication.
c. Standard of care bloods can be used as screening bloods, if taken within +/- 2 weeks from the screening visit. Participants without virology screening bloods will still be eligible to take part. Patients known to be positive for Hepatitis B, C or HIV will be excluded from study entry.
d. Optional biopsies are offered to all non-surgical patients prior to radiotherapy, and to all patients where progression has been identified. Optional biopsy should ideally take place **after** research scanning to minimize any biopsy-related changes. Archival biopsy may also be collected.
e. Tissue swabs should be taken from the tumour site (preferred) or cheek mucosa (where tumour is not accessible). Post surgery, swabs taken from the cheek mucosa will be sufficient and should be collected at all tissue swabbing timepoints.
f. Research blood samples to be coordinated with regular clinical appointments and/or routine blood sampling to minimize travelling and inconvenience to patient.
g. All the participants taking part in the optional research imaging (MRI +/- MRE) will have confirmation from the study radiologist if the tumour size and location is MRE assessable as per Imaging Manual. Mid-radiotherapy research imaging to be performed in non-surgical cohort.
h. 24 months or progressive disease whichever comes first.
i. Optional Research MRI (+/- MRE). MRE scans are only applicable to those sites that can perform them
